# Supplementary material for: Are carbon emissions trading and green financial instruments synergistic? -Comprehensive quantitative research based on content analysis
Source: PLoS One. 2024 Mar 7;19(3):e0298601. doi: 10.1371/journal.pone.0298601 (PMC10919601; doi:10.1371/journal.pone.0298601)
Supplement: S1 Data — (ZIP) [file pone.0298601.s001.zip › Data/地级市Z_F工作报告.zip!/║■▒▒/╩«╤▀╩╨/└┤╫╘╣1⁄2╓┌║┼í╛┬φ┐╦╩2╛▌═°í┐_2008.docx]

各位代表：

　　现在，我代表市人民政府向大会作工作报告，请予审议，并请各位政协委员和其他列席会议的同志提出意见。

　　一、2007年政府工作回顾

　　2007年，在市委的正确领导下，全市上下深入贯彻落实科学发展观，克难奋进，顽强拼搏，抢抓机遇，加快发展，全市经济发展呈现速度加快、效益提高、活力和后劲增强的良好态势，社会事业全面进步，人民生活进一步改善，市三届人大三次会议确定的主要预期目标全面超额完成。预计全市生产总值突破400亿元，同比增长15%以上。

　　（一）经济发展实现新跨越。

　　农村经济持续发展。扎实推进新农村建设，奋力抗灾自救，农村经济保持了良好的发展势头。全市第一产业实现增加值44亿元，增长4%；农民人均纯收入增加246元。粮油产量创历史最高水平，畜牧业全面稳定增长。农业产业化加速推进，新增基地20万亩，市级以上重点龙头企业达69家，10个农产品被认定为省级著名商标和名牌产品。劳务经济继续壮大，外出务工农民达到63万人，务工收入达30亿元。扶贫开发深入推进，不得温饱人口减少4万人，低收入人口减少5万人，搬迁扶贫1.18万人。县域经济快速发展，五县一市实现生产总值155亿元，增长11%。

　　工业经济高速增长。在全力支持服务东风公司的同时，大力推进"双亿"工程，着力打造工业园区平台，培植壮大地方工业实力，工业经济速度、效益同步高速增长。全市工业总产值突破600亿元，增长35%，实现利润24亿元，增长44%，新增规模以上工业企业53家。重点行业快速增长，汽车产业增长45%，电子信息产业增长33%，化工产业增长30%。"双亿"工程企业发展势头强劲，33家企业实现产值105亿元，增长38%，新增产值过亿元的企业达7家。工业园区经济发展迅速，8个重点工业园区增加值增长37%。品牌建设初见成效，新增国家免检产品2个，"通达牌"消声器被评为中国名牌产品。

　　服务业快速增长。第三产业实现增加值166亿元，增长12%。旅游产业长足发展，全市接待国内外游客760万人次，实现旅游总收入31.6亿元，创汇1727万美元，分别增长14%、12.8%和28.9%，武当山接待游客首次突破百万人次大关。城乡消费日趋旺盛，城镇居民人均可支配收入8870元，增长15%；实现社会消费品零售总额170亿元，增长17.5%；新增农村超市388家、农资放心店370家。

　　财政金融运行平稳。实现财政收入37.5亿元，增长10%，其中一般预算收入15.6亿元，增长18.1%。金融机构各项存款余额475亿元，比年初增加39亿元；各项贷款余额205亿元，比年初增加29亿元。

　　（二）项目建设取得新成效。

　　重大项目进展顺利。完成全社会固定资产投资145亿元，增长24%。总投资44亿元的潘口电站正式开工建设，丹江大坝加高工程投资过半，三里坪电站成功截流，50万伏十堰输变电工程完成线路铺设。城区万亩山地整理项目已经动工。十漫高速公路建成通车，襄渝铁路二线进展顺利，十天高速、郧十高速前期工作有序推进，柳树垭隧道已经贯通，五条岭隧道开工建设，改造干线公路189公里，新建省际出口路及经济断头路180公里。东风中客形成5000辆生产能力，东风渝安形成8万辆微型车生产能力，双星东风达到300万套轮胎生产能力，郧县、房县等四个百万吨干法水泥项目开工建设，福润公司3000万只肉鸡加工项目投产运营。

　　农村基础建设步伐加快。新修通村水泥路2700公里，新建农村五级客运站7个，候车亭、招呼站414个。维修水库45处，加固各类堤防172公里，恢复水毁农田1.6万亩。建设农村供水工程112处，解决11.7万人饮水安全问题，治理水土流失面积180平方公里。验收土地复垦整理项目159个，净增耕地1.37万亩。新建农村沼气2.4万户，推广节柴灶4.1万座。

　　城镇建设力度加大。推进"百镇千村"示范工程，全市城镇化率达到39%。武当路、浙江路、凯旋大道一期建成通车，丹江路、许白路、花果路改造进展顺利。鄂西北小商品市场投入使用，堰中蔬菜批发市场二期工程即将建成。重手整治市容市貌，推进环卫体制改革，建立了市容环境卫生长效机制，基本形成了市、区、街办、社区四级联动、各负其责的市容环卫管理新格局。郧西县上津镇被评为中国历史文化名镇。

　　（三）改革开放取得新突破。

　　各项改革深入推进。农村综合配套改革不断深化，农村财务管理逐步规范，乡财县管、以钱养事机制初步建立，共化解村级债务5146万元；土地二轮延包工作基本完成，集体林权制度改革试点有序推进。国企改制成果进一步巩固，部分遗留问题得到解决，国有企业改制面达97%，以东风轮胎为代表的一批困难企业焕发出新的活力。财政改革步伐加快，市级部门预算全部推进到位，国库集中支付改革逐步向乡镇一级延伸，非税收入直接征收面达90%以上。

　　对外开放水平不断提高。成功举办深圳投资推介会，参加香港湖北周、中部投资博览会、西洽会、华创会等招商活动，全市实际利用外资3900万美元，增长12.7%；利用市外境内资金31.7亿元，增长20%。全市进出口企业发展到210家，完成外贸出口6000万美元，增长53%。汉江集团、先锋模具两家企业在境外投资810万美元，黄龙电厂承接海外电站管理，外派劳务增长25%。

　　（四）社会事业取得新进步。

　　科教事业稳步发展。专利申请增长30%，高新技术企业达到86家，产值过亿的高新技术企业11家，黄姜清洁生产等3个项目纳入国家科技支撑计划和星火计划，山地农业综合开发集成技术获省政府科技进步一等奖，省胚胎干细胞研究重点实验室落户十堰，我市被批准为全国知识产权试点城市。全面推行农村义务教育经费保障机制，免除农村义务教育学生学杂费，免费为14.6万名贫困生发放教科书，为3.87万名贫困寄宿生补助生活费，新建和维修农村校舍12万平方米，全市小学和初中适龄儿童入学率分别达到100%和98.2%，"两基"工作顺利通过国家验收，高校迁建、扩建工程顺利推进。

　　文体事业蓬勃发展。市博物馆建成开馆，吕家河民歌等7个非物质文化遗产被列入省级保护名录，淹没区文物抢救工作进展顺利，出土珍贵文物1.4万件。健身中心、羽毛球馆投入使用，改造21个乡镇综合文化站，完成100个行政村健身工程。成功举办武当武术国际发展战略研讨会，央视"心连心"艺术团来库区慰问演出。新铺设光缆155公里，架设电缆1286公里，广播电视"村村通"和央视无线覆盖率达到97%，完成16万户卫星广播电视的转星调整工作。

　　医疗卫生服务体系逐步健全。新型农村合作医疗实现全覆盖，参合人口达209.5万人，参合率86%；改造14个乡镇卫生院，新建353个村卫生室。城区和县市城关各类卫生机构达129个，覆盖城镇人口97.4万人。惠民医院投入运营。筹措医疗救助资金580万元，救助困难群众11.2万人次。开展了食品、药品专项整治工作，保障了人民群众饮食和用药安全。继续稳定低生育水平，人口出生率控制在省定目标以内。

　　（五）和谐十堰建设开创新局面。

　　就业和社会保障进一步加强。新增就业3.65万人，下岗失业人员再就业1.9万人。城市低保对象实现应保尽保，月补助标准逐步提高；全面启动农村低保，11.9万农村特困救助对象由临时性救助转入制度性保障；1.04万失地困难农民纳入城乡低保救助。社会保险扩面新增6.34万人，各项社会保险金按时足额发放，企业离退休人员增资政策落实到位。建立了城区廉租住房保障制度，发放补贴60万元；改造农村危房2439户。落实救灾资金3000余万元，受灾群众生活得到有效保障；投入倒房恢复重建资金5000万元，7000多倒房户全部搬入新居。

[FS:PAGE]

　　生态建设成效显著。水源区污染防治项目全面启动，泗河、竹山等5个污水处理厂开工建设，8个污水、垃圾处理项目获国家发改委审查批准。大力开展环保专项治理，关停69家黄姜加工企业、5家小水泥企业，关停或限期治理36家小造纸、小电镀企业。狠抓节能减排工作，万元生产总值能耗下降4%，主要污染物排放总量控制在省定目标以内。完成造林41万亩，森林覆盖率达52%。城镇各类饮用水源水质达标率100%，空气质量在二级标准以上的天数占全年的96.4%。

　　社会秩序保持稳定。加强社会治安防控体系建设，完善公共突发事件应急机制，及时排查、整改重大安全隐患，各类事故下降31.7%。高度重视信访工作，信访总人次、进京非正常上访人次分别下降32.6%、53.3%。市公安局被评为全国公安信访工作先进集体。

　　（六）民主法制和精神文明建设取得新进展。

　　依法行政水平不断提高。市政府自觉接受人大法律、工作监督和政协民主监督，定期向人大报告工作、向政协通报情况，重大决策征求民主党派、党外人士、人民团体意见，全年办理人大代表建议163件，办理政协委员提案358件，办复率100%。推行政务公开，开展作风建设专题教育活动和民主评议政风行风工作，123家公用事业单位实行了办事公开，市长热线受理群众反映的问题2.1万条，反馈率达100%。依法依规对行政事业单位资产、"吃空饷"、临时用工和福利企业规范管理等情况进行了全面清理，并相应出台了加强规范性管理的改革措施。推进行政执法责任制，行政复议案件结案率92.3%，追究行政执法违法和错案4件。受理各类行政审批事项2.2万件，办结率99%。推进"五五"普法，积极开展法律援助活动。

　　精神文明建设向纵深推进。加强公民素质教育，加快"文明十堰"建设步伐，积极开展文明社区、文明单位、文明农户等系列创建活动，建设文明新村试点121个，涌现了91个省级文明单位。深化全民国防教育，组织开展国防动员演练和民兵阅兵比武活动，深入开展"双拥"活动，全面落实拥军优抚安置政策，积极支持部队和国防建设，实现全国"双拥模范城"五连冠。

　　这里着重报告去年"十件实事"进展情况。一年来，市政府克服各种困难，确保了十件实事顺利推进，解决了一批市民关心的热点难点问题。计划当年完成的新增就业、减少贫困人口、廉租住房保障、失地困难农民救助、农村危房改造、饮水安全、通村公路、农村合作医疗、社区卫生服务、惠民医院试点、堰中蔬菜批发市场、农村超市和农资放心店等实事全部完成或超额完成目标任务；跨年度的城郊居民饮水、新建初中和扩建柳林小学、污水处理厂建设、50万伏输变电工程、天然气利用等实事都取得实质性进展。

　　在全市经济社会建设中，人事、审计、统计、物价、外事侨务、民族宗教、档案、地方志、邮政、地震、气象、水文、人防、对台、老龄、残联等工作都取得新的成绩。

　　各位代表，上述成绩的取得，是市委正确领导的结果，是市人大、市政协全力支持的结果，是全市人民共同奋斗的结果。我代表市政府，向所有参与、支持、关心十堰发展的同志们、朋友们表示衷心的感谢和崇高的敬意!

　　在总结成绩的同时，还要看到十堰最大的实际仍然是发展不够，在前进道路上还面临不少困难和问题。比如：农村基础依然薄弱，农民收入稳定增长的渠道不宽，农村生产生活条件还需进一步改善；工业化水平不高，长期形成的结构性矛盾仍未改变，汽车主导产业发展的不确定性因素较多，工业经济增长的持续性和稳定性较差；承接国内外产业转移的力度不够，重大投资项目较少，市场主体发育不够，县域经济薄弱的状况还没有根本好转；生态环境建设历史欠账多，环境保护压力大，节能减排任务重；涉及人民群众切身利益的问题还比较突出，劳动就业、社会保障、教育卫生、居民住房、安全生产、社会治安等方面都还有很多问题亟待解决；国企改制、土地征用、城镇拆迁、移民安置等方面也仍然存在着潜在的不稳定因素，促进社会和谐任务还相当艰巨；政府自身改革和建设还需要进一步加强，一些部门办事效率不高、执行力不强，有些政府工作人员作风不实，一些领域腐败问题仍然比较严重，等等。对于这些困难和问题，我们将在今后的工作中采取有效措施认真加以解决。

　　二、2008年工作思路和主要任务

　　今年是全面贯彻落实党的十七大精神的开局之年，也是实施"十一五"规划承上启下的一年，做好今年的工作，具有十分重要的意义。新的一年，我市发展面临难得的机遇和有利条件：国家促进中部地区崛起、推进新阶段扶贫开发、划分主体功能区并建立生态补偿机制、出台丹江库区经济社会发展规划、实施"两比照、一支持"政策，我市进入新一轮国家区域性优惠政策叠加期；南水北调中线工程加快推进，库区水污染防治和水土保持规划全面实施，移民内安外迁工作试点启动，襄渝铁路二线全线铺轨，潘口电站建设全面铺开，我市进入新一轮投资高峰期；东风公司带动力将进一步显现，"双亿"工程企业实力将进一步增强，改制企业发展活力将进一步提高，消费旺盛和出口增长的动力将进一步彰显，经济自主增长潜能将进一步释放，我市进入新一轮加快发展期。我们要紧紧抓住难得的发展机遇，坚定信心，乘势而上，扎实工作，努力实现我市经济社会更好更快发展。

　　今年我市经济社会发展的总体要求是：高举中国特色社会主义伟大旗帜，以邓小平理论和"三个代表"重要思想为指导，全面贯彻党的十七大和省第九次党代会精神，深入贯彻落实科学发展观，以大开放为先导，大力实施工业兴市、生态立市和人才强市战略，着力调整经济结构和提高经济增长质量，积极推进体制改革和自主创新，切实加强节能减排和生态环境保护，更加重视改善民生和促进社会和谐，努力优化发展环境，不断开创经济社会发展的新局面。对今年全市经济社会发展主要奋斗目标的建议是：生产总值增长10%，地方一般预算收入增长12%，全社会固定资产投资增长18%，社会消费品零售总额增长15%，利用外资增长15%，外贸出口总额增长15%，居民消费价格总水平控制在4.8%以内，城镇居民人均可支配收入增长8%，农民人均纯收入增长8%，登记失业率控制在4.5%以内，人口自然增长率控制在6‰以内，万元生产总值能耗、主要污染物排放总量控制在省定目标以内。保证以上目标的实现，要重点抓好八个方面的工作。

　　（一）大力发展现代农业，扎实推进新农村建设。

　　做好"三农"工作，对稳定经济社会发展大局具有特殊意义。我们必须增强做好"三农"工作的紧迫感，把解决好"三农"问题作为工作重中之重。以农民增收为主线，切实加大统筹城乡发展力度，积极探索山区农业现代化的新路子。

　　推进农业产业化。加强市级农业研发中心建设，改造、新建柑桔、茶叶、烟叶等特色产业基地20万亩，推进标准化示范区工作，加快农业标准化、信息化、机械化步伐；建设高效经济林基地20万亩，加快高速公路沿线产业带建设。积极发展养殖小区，推进规模经营，加强动物疫病防控，促进畜牧业大发展。加大龙头企业扶持力度，引导发展农民专业合作社，着力打造有机农业品牌，力争新增省级农业产业化龙头企业3家以上，新认证农产品品牌20个左右。

　　积极发展劳务经济。加强服务体系建设，开展转移培训，提高组织化输出水平，力争全年务工收入增长10%以上。扩大外派劳务规模。多渠道解决农民工社会保障问题，推进基本医疗保险和大病医疗救助，在农民工集中、风险高的行业全面推行工伤保险。

　　深入开展扶贫开发。全面完成去年的111个重点贫困村建设任务，启动今年的109个重点村建设，抓好24个重点老区村整村推进工作，开展扶贫培训2.65万人。推进城区农村扶贫开发，实现村通水泥路、广电、通信全覆盖的目标。

[FS:PAGE]

　　加强农村基础设施建设。加强耕地保护，实现耕地总量动态平衡。加快实施病险水库加固，抓好灌区配套工程建设，新增节水灌溉面积3.6万亩。建设沼气示范村30个，治理水土流失面积200平方公里。继续实施"百镇千村"示范工程，加快重点村镇基础设施建设。

　　进一步深化农村改革。坚持农村基本经营制度，稳定和完善土地承包关系，按照依法自愿有偿原则，健全土地承包经营权流转市场，支持发展多种形式的规模经营。巩固农村税费改革成果，积极化解乡村债务。扎实开展集体林权改革，确权发证率达到80%以上。

　　（二）强化产业支撑，全力做强做大工业经济。

　　以汽车产业为重点，以开发区、工业园区为载体，以"双亿"工程为抓手，坚持汽车工业与地方特色工业两手抓，城区工业与县域工业两手抓，整车生产与汽配生产两手抓，着力延伸产业链条，壮大产业规模，发展产业集群，全面推进工业结构调整和优化升级，不断提高工业经济运行质量和效益。

　　继续抓好"双亿"工程。全面落实《支持城区加快新型工业化发展若干政策规定》，坚持领导联系企业制度，及时解决企业面临的困难和问题。安排专项扶持资金1500万元，对列入"双亿"工程的企业给予支持补助。加快三环专汽3万辆、双星东风轮胎600万套、东风渝安10万辆、金鼎纺织10万锭等扩能项目的建设步伐，全力推进"双亿"工程在建项目建设，力争"双亿"工程企业发展到50家，实现产值150亿元。

　　着力发展园区工业。加强园区规划和基础设施建设，支持十堰经济开发区、东城和西城开发区、县市工业园区发展，鼓励企业向开发区、工业园区集中，生产要素向优势产业集中，不断提升凝聚资源、聚合产业的能力。继续推进山地整理，力争开发建设用地5000亩。实行"一区多园"，加快普林二期、东风创业孵化园、温州汽配等园区建设步伐，推进万向工业园的项目入驻对接工作。

　　大力发展汽车工业。全力服务东风公司，支持其重大项目建设，争取更多项目落户十堰。大力发展整车和零部件生产，重点抓好专用车、改装车、微型车和低速运输车生产。建设国家级汽车零部件检测中心，鼓励模具、冶炼、铸造等上游产业发展，支持贸易、服务等下游产业发展，整合汽车产业资源，引导企业专业化分工协作，发展和延伸产业链，壮大汽车产业集群。

　　加快发展特色工业。加快水电开发步伐，抓好潘口、周家垸、三里坪等电站建设，力争孤山、龙背湾、小漩、白沙等水电项目早日获准开工，全面完成50万伏十堰输变电工程建设，做好120万千瓦热电联供项目的争取工作。推进鄂西北食品加工工业园、四个新型干法水泥等项目建设，壮大医药化工、绿色食品等产业实力，加快冶金、燃化、建材、轻纺等传统工业和信息产业发展步伐。全面落实促进县域经济发展的各项政策，推进城区与县市产业融合，支持县域工业加快发展。

　　实施中小企业成长工程。加大对中小企业扶持力度，健全服务体系，搭建加快发展的平台。完善担保机制，支持有条件的企业通过多种方式直接融资，支持企业通过联营、参股、嫁接等合作方式做大，引导企业通过建立现代企业制度做强。支持专业技术人员、大中专毕业生等创办实体，鼓励十堰籍企业家和成功人士回乡创业，力争全市新增各类企业1000家以上，其中规模以上企业100家以上。

　　提高企业自主创新能力。加大科技投入，优化科技资源配置，健全激励和风险投资机制，加快创新型城市建设。大力推进企业技术创新引导工程，重点支持25家进入全省"五个一百"工程的企业。实施汽车电子、装备制造、生物医药、节能环保、黄姜清洁生产等自主创新专项，推进省级工程技术中心建设。支持各类科技中介服务机构发展，促进科技成果转化，发明专利申请增长25%以上。继续实施精品名牌战略，力争实现新的突破。

　　（三）培育消费热点，突破性发展第三产业。

　　大力发展旅游业。提升武当山、汽车城、水源区三大品牌，启动实施库区生态旅游发展规划，加大城市工业旅游资源开发力度，加快五龙景区、玉虚宫、太极湖新区等重点项目建设进度。推进五龙河、白马山、"双野"、十八里长峡等景区建设。加强区域旅游协作，广泛开展旅游促销，力争全年接待国内外游客突破900万人次，实现旅游总收入40亿元。

　　加快城乡市场体系建设。支持中国（十堰）汽配城扩大规模，支持建设好风神、汉江、房陵等物流园区。做好天津路、重庆路沿线发展规划，打造火车站商贸经济圈。加快云燕农博园建设，完成农村合作超市和农资放心店各100个。

　　着力扩大消费。引进培育大型流通企业集团，支持连锁超市、仓储批发及便利店等新业态发展，鼓励企业开发农村消费市场。积极推动消费结构升级，加快发展电子商务、健身、物业、信息、社区服务等产业，支持营销策划、投资顾问、咨询服务等产业发展。

　　（四）扩大对外开放，培植壮大开放型经济。

　　提高招商引资水平。坚持以大开放为先导，以招商引资为重点，努力提高对外开放水平。坚持引进外资与引进内资并重，更加积极主动地承接沿海地区产业转移，加强与武汉城市圈等经济协作区的对接工作。要创新招商方式，强化企业招商主体地位，突出汽车、能源、医药、轻纺、农产品加工等重点领域，积极组织各类招商活动。注重招商实效，搞好项目后续服务，不断提高项目履约率和资金到位率。着力引进战略投资伙伴，争取利用外资过5000万美元、市外境内资金达到30亿元以上。

　　转变外贸增长方式。大力优化出口商品结构，着力培育出口品牌，支持东风汽车进出口公司、双星轮胎、东风实业、益民铜材等企业开拓国际市场，扩大高新技术、机电产品出口规模。加快食用菌、五倍子、桑蚕等农特产品出口基地建设，提高农产品出口竞争力。支持有实力的企业对外承揽工程、开展技术合作，发展服务贸易。

　　（五）做好财税工作，提高金融服务能力。

　　加强财税工作。继续做好主体功能区补偿、中部老工业城市试点、中央转移支付等政策性资金的争取工作。综合利用国债、税收、贴息、担保等扶持方式，培植壮大财源。加强税收征管，确保财政收入稳定增长。优化支出结构，把财力保障的重点，向社会事业、基础设施建设和公共事业发展倾斜，向扩大就业、完善社会保障体系和改善困难群众生活倾斜，向保障公共安全、维护社会和谐稳定倾斜，使全体人民共享发展成果。深化财政改革，推行预算单位公务卡，实现财政管理精细化。完善国有资产管理体制，建立规范的政府出资人制度和国有资本经营预算制度。

　　完善金融服务。发挥金融支持地方发展的作用，加强银企合作，引导各金融机构加大对城市建设、项目建设和新农村建设的支持力度。加强社会信用体系建设，改善金融发展环境。支持商业银行改革，稳妥推进农村信用社改革，支持股份制银行和外资银行在我市设立贷款子公司。引导金融机构优化信贷结构，开发支持企业发展、适应农村需要的金融产品，推进金融服务创新。

　　（六）开展"三城"联创，增强城市竞争力。

　　以创建国家卫生城市、全国文明城市、国家环保模范城市为目标，加强城市公共基础设施建设，完善城市服务功能，提高城市管理水平，提升城市形象和品位。

　　改善交通条件。着力构建对外大交通、对内大循环的交通网络。积极服务襄渝铁路二线建设，力争年内建成通车；做好十天高速、郧十高速公路的前期工作。建设209国道杜阳段，推进汉江二桥、习均大桥和五条岭、雷峰垭、龙王垭、土地岭等隧道建设，加快国省道危桥改造和安保工程建设步伐，整治汉江航道。

　　加强城市建设和管理。认真贯彻落实《城乡规划法》，精心组织新一轮城市总体规划修编工作，切实做好控制性详规及专项规划。加快城市道路建设，续建凯旋大道、丹江路、许白路，改造十堰二桥、江苏路口。完善城市交通设施，建设十堰高速客运站，推进公交站廊及公共停车场改造。推进城区污水处理厂和垃圾处理场建设，新建一批垃圾中转站和公厕。加强社区建设，提高社区公共服务水平。健全城市长效管理机制，进一步改善市容环境，争创国家卫生城市。

[FS:PAGE]

　　建设生态文明。巩固国家生态示范区建设成果，争创国家环保模范城市。建立环境与发展综合决策机制，实行环保问责制和一票否决制。继续开展环保专项整治，支持循环经济试点，提高工业"三废"综合利用率。全面实施节能减排工程。积极实施库区水污染防治和水土保持规划，加快生态产业、污水和垃圾处理等规划项目建设步伐，加强农业面源污染治理，确保饮用水质保持在国家二级标准以上。巩固退耕还林成果，推进天然林保护工程和长防林、生态公益林建设，完成人工造林30万亩。

　　深化文明创建。加强公民道德教育，积极开展群众性精神文明创建活动，提高市民文明素质和城市文明程度。以社区创建为重点，推进城市文明创建活动，确保省级文明城市"五连冠"，争创全国文明城市。以文明新村为重点，推进农村文明创建活动，力争涌现一批文明村镇。加强全民国防教育，巩固军政军民团结，全面落实国防动员各项任务，加强人民防空和民兵预备役建设，巩固"双拥模范城"创建成果。

　　（七）注重改善民生，全面推进社会建设。

　　优先发展教育事业。深化教育改革，合理调整教育结构，促进义务教育均衡发展，加强职业教育，提高高等教育质量。支持高等院校扩建，鼓励和规范社会力量兴办教育。进一步完善农村义务教育经费保障机制，推进农村寄宿制学校建设，提高贫困学生生活补助费标准。从今年秋季开学起，免除城市义务教育学杂费，保障城乡所有适龄儿童平等接受义务教育。做好农村和城市低保家庭中职生资助工作。

　　大力发展文体事业。积极发展文化体育、广播电视、新闻出版、科学普及事业，坚持正确导向，弘扬社会正气。推进文化体制改革，积极开发文化产品，培植文化品牌，繁荣文化市场。加强基础文化设施和基层文化服务体系建设，抓好乡镇文化站、农村电影放映、乡戏进城、农家书屋等重点工程。重视非物质文化遗产保护，继续抓好淹没区文物抢救发掘。加强对外文化交流，申办"第三届世界传统武术节"，办好武当国际旅游节。推进广播电视数字化进程。广泛开展全民健身运动。

　　加快发展医疗卫生事业。提高应对突发公共卫生事件的能力，建设覆盖城乡居民的基本卫生保健体系，不断提升区域医疗中心地位。全面推进新型农村合作医疗，实现以县（市、区）为单位全覆盖。完善社区卫生服务体系，推行双向转诊制度，提高居民就诊率和满意率。坚持药品集中招标采购，控制医疗费用过快增长。建立计划生育利益导向机制，稳定低生育水平，提高出生人口素质，遏制违法违纪生育反弹和出生人口性别比失衡的势头。

　　千方百计扩大就业。多渠道开发就业岗位，大力发展就业容量大的产业，鼓励自主创业、自谋职业和灵活就业，大力推进城乡统筹就业，做好农村劳动力转移培训工作，认真解决体制转轨遗留的下岗失业人员再就业问题和改制重组、关闭破产企业的职工安置问题。完善面向所有困难群众的就业援助制度，及时帮助零就业家庭解决就业困难。规范和协调劳动关系，落实最低工资标准，健全企业职工工资正常增长机制和支付保障机制。加大劳动保障监察执法力度，依法保护劳动者合法权益。

　　健全社会保障体系。扩大社会保险覆盖面，新增参保6万人。巩固完善城镇低保制度，建立城镇居民基本生活消费品价格变动应急救助预案制度、动态补贴制度、临时救助制度和保障标准自然增长机制。全面启动农村低保，逐步扩大保障覆盖面。认真做好农村五保供养工作，集中供养、分散供养标准分别提高到1500元和1000元，建设城市养老社会化服务示范点。加强住房保障体系建设，完善廉租房制度。完善医疗救助制度，加大流浪未成年儿童救助力度。发展慈善、老龄、残疾人和红十字事业。

　　认真做好移民工作。全力服务南水北调中线工程建设，高质量完成1万人外迁安置试点任务，适时启动内迁安置试点工作，为下一步大规模移民安置积累经验。大力支持潘口水电站建设，保证移民安置任务按期落实，确保10月份大坝截流。全面落实移民政策，及时解决移民生产、生活中的困难和问题，保障移民的合法权益。

　　全力维护社会稳定。完善应急管理机制，提高突发事件的快速反应和有效处置能力。深入推进"平安十堰"建设，依法打击各类违法犯罪活动，保障人民生命财产安全。落实安全生产责任制，有效预防重特大事故发生。健全信访工作机制，注重从源头上化解矛盾，确保各类上访大幅下降。解决好改制企业遗留问题。加强食品药品监管，保障人民群众健康安全。强化市场、物价监管，努力保持主要消费品和服务价格基本稳定，完善和落实对低收入群众的补助办法。

　　（八）强化自身建设，着力提升政府形象。

　　加强民主建设。主动接受人大监督、政协监督、社会监督。完善重大事项专家咨询、集体决策、社会公示和听证制度，提高民主决策水平。推行政务公开，完善厂务公开、村务公开和公共事业单位办事公开制度。发挥市长热线、行风评议热线、政府门户网站等媒介作用，继续开展民主评议政风行风工作，畅通民情民意反映渠道，保障群众的知情权、参与权、表达权和监督权。加强基层民主建设，开展第七届村（居）委会换届选举工作。

　　坚持依法行政。全面落实《依法行政实施纲要》，进一步规范政府行为，严格按照法定权限和程序行使权力、履行职责。健全行政复议、法律顾问等制度，推进政府工作法治化、制度化和规范化。完善规范性文件定期清理和修改废止制度。深入开展"五五普法"宣传教育活动。

　　建设服务型政府。落实国家行政体制改革要求，完善公共服务体系，强化社会管理和公共服务。进一步减少和规范行政审批，完善行政服务中心功能，推行电子政务，提高行政效率。大力优化发展环境，推进管理创新、服务创新，提升公务员队伍素质，增强服务意识和责任意识，更好地为基层、企业和社会公众服务。在关注民生的同时，做好煤、电、油、运等重要生产资料的组织协调工作，保证企业生产需要。

　　强化勤政廉政。进一步完善预防和惩治腐败体系，从源头上预防腐败。坚持从严治政，完善绩效考核，加强效能监察，确保政令畅通。完善机关工作人员行政过错责任追究办法，强化行政问责，严肃查处有令不行、有禁不止、作风不实等问题。坚持勤俭节约，反对铺张浪费，切实降低行政成本。突出抓好重点部门、关键岗位的监督，支持监察、审计部门依法独立履行监督职责。严格执行工程建设招投标、经营性土地使用权出让和政府采购等制度，深入开展治理商业贿赂专项工作，严肃查处腐败行为，坚决纠正损害群众利益的不正之风。

　　各位代表，今年市政府将继续办好十件实事，着力解决群众关心、社会关注的热点难点问题。（1）城镇新增就业3.5万人，组织农村劳动力转移就业新增6万人。（2）完成农村扶贫搬迁2800户，减少贫困人口8万人。（3）实施农村寄宿制学校建设工程，对农村200所中小学实施改水、改厕。（4）建设通村水泥路1500公里，将已建成的7500公里村级水泥路纳入养护范围。（5）解决农村群众饮水安全10万人，新建农村"一建三改"沼气2万户、节柴灶4万座。（6）将所有农民纳入新型农村合作医疗制度覆盖范围，支持20个乡镇卫生院和200个村卫生室完善基础设施。（7）纳入城乡低保人口达到20万人以上。（8）开发建设城区经济适用房10万平方米，改造农村危房2000户。（9）完善社区服务功能，实施社区亮化工程，改造城区背街小巷道路6万平方米。（10）开放牛头山森林公园，实施艳湖露天游泳池改造工程，建设上海路。

　　各位代表，做好今年的工作，对于我市顺利实现"十一五"规划，全面建设小康社会，具有十分重要的意义。我们要在市委的正确领导下，团结拼搏，开拓进取，扎实工作，全面完成各项目标任务，为迎接十堰更加美好的明天而努力奋斗！
